# PRESIDENTIAL POLITICS AND THE FORTITUDE FACTOR

**DOI:** 10.1093/geroni/igae098.2094

**Published:** 2024-12-31

**Authors:** Brian Lindberg

**Affiliations:** Gerontological Society of America, Washington, District of Columbia, United States

## Abstract

Education, engagement, advocacy, and coalition building are key elements of GSA’s ongoing work in the nation’s capital. Election years provide both opportunities and challenges for working in the legislative and regulatory processes, and GSA staff and our members across the country have become effective in sharing the research, policy development, and practice experiences with policymakers in DC. Although the field may face challenges in “advocacy cohesion,” this presentation will include examples of the continued strength of aging advocates in the face of ageism and generational competition for resources. GSA’s intergenerational approach to engagement and our recent policy initiatives will be discussed. The presentation will include examples of the strength of aging advocacy organizations and their ability to successfully protect federal programs facing proposed cuts or elimination and securing billions of dollars in additional resources during the COVID-19 pandemic. While the timing is not good for large new entitlement programs, coalitions have been successful in improving benefits such as home delivered meals, tackling the social isolation crisis, providing resources for caregivers, and reducing the cost of Medicare prescription drugs and more. GSA is often providing the evidence to support these positive steps.

